# Epidemiology of Pathogenic Retroviruses and Domestic Cat Hepadnavirus in Community and Client-Owned Cats in Hong Kong

**DOI:** 10.3390/v16020167

**Published:** 2024-01-23

**Authors:** Julia A. Beatty, Yan Ru Choi, Omid Nekouei, Fiona. M. Woodhouse, Jane. J. Gray, Regina Hofmann-Lehmann, Vanessa R. Barrs

**Affiliations:** 1 Department of Veterinary Clinical Sciences, Jockey Club College of Veterinary Medicine and Life Sciences, City University of Hong Kong, Hong Kong SAR, China; julia.beatty@cityu.edu.hk (J.A.B.); yrchoi@cityu.edu.hk (Y.R.C.); 2 Centre for Animal Health and Welfare, City University of Hong Kong, Hong Kong SAR, China; 3 Department of Infectious Diseases and Public Health, Jockey Club College of Veterinary Medicine and Life Sciences, City University of Hong Kong, Hong Kong SAR, China; omid.nekouei@cityu.edu.hk; 4 The Society for the Prevention of Cruelty to Animals, Wan Chai, Hong Kong SAR, China; fiona.woodhouse@spca.org.hk (F.M.W.); jane.gray@spca.org.hk (J.J.G.); 5 Clinical Laboratory, Department of Clinical Diagnostics and Services, Center for Clinical Studies, Vetsuisse Faculty, University of Zurich, 8057 Zurich, Switzerland

**Keywords:** cat, feline, felid, retrovirus, immunodeficiency, leukaemia, virus, hepatitis-B, hepadnavirus

## Abstract

Understanding the local epidemiology of feline leukaemia virus (FeLV) and feline immunodeficiency virus (FIV) in Hong Kong will inform retrovirus prevention strategies. Domestic cat hepadnavirus (DCH), a novel hepatitis-B-like virus, is commonly detected among client-owned cats in Hong Kong, but community cats have not been studied. The aims of this study were to investigate the frequency and potential risk factors for (i) FeLV and FIV among community and client-owned cats and (ii) perform molecular detection of DCH among community cats in Hong Kong. Blood samples from 713 cats were obtained from client-owned (n = 415, residual diagnostic) and community cats (n = 298, at trap-neuter-return). Point-of-care (POC) testing for FeLV antigen and feline immunodeficiency virus (FIV) anti-p15 and p24 antibodies was performed. FeLV-positive samples were progressed to p27 sandwich enzyme-linked immunosorbent assay. Whole blood DNA was tested with qPCRs for FeLV U3 and gag, and nested PCRs where additional information was required. DCH qPCR was performed on a subset of community cats (n = 193). A single, regressive, FeLV infection was detected in a client-owned cat (1/415 FeLV U3 qPCR positive, 0.2%, 95% CI 0.0–1.3%). Five/415 client-owned cats tested presumably false FeLV-antigen positive (qPCR negative). No markers of FeLV infection were detected in community cats (0/298; 0%). FIV seroprevalence was much higher in community cats (46/298, 15.4%) than in client-owned cats (13/415, 3.1%) (*p* < 0.001). Mixed breed was a risk factor for FIV infection in client-owned cats. Neither sex nor age were associated with FIV infection. DCH DNA was detected in 34/193 (17.6%) community cats (median viral load 6.32 × 10^3^ copies/reaction). FeLV infection is rare in Hong Kong, negatively impacting the positive predictive value of diagnostic tests. FeLV-antigen testing remains the screening test of choice, but confirmation of a positive result using FeLV qPCR is essential. FIV infection is common in community cats and the absence of a sex predisposition, seen previously in cats managed similarly, raises questions about virus-transmission dynamics in these groups. DCH infection is very common in Hong Kong, both in client-owned and community cats, highlighting the importance of understanding the pathogenic potential of this virus for cats.

## 1. Introduction

Two viruses in the family *Retroviridae*, namely feline leukaemia virus (FeLV, genus *Gammaretrovirus*), and feline immunodeficiency virus (FIV, genus *Lentivirus*) are important pathogens of domestic cats. When FeLV evades host immune control, progressive infection is established causing profound bone marrow suppression, lymphoma and other cancers, and a significantly reduced lifespan [1]. FIV infection causes a progressive immune dysfunction which may or may not manifest clinically but, in contrast to FeLV, life-threatening outcomes associated with FIV infection are less commonly reported [2].

Patient testing for FeLV and FIV is frequently indicated because of the non-specific nature of clinical signs associated with infection and the global distribution of these viruses. Screening tests that detect FeLV p27 antigen or anti-FIV antibodies are commonly combined in point-of-care (POC) assays. Interpretation of test results requires the integration of data related to the test, the patient and the prevalence of the virus, with the latter varying widely between regions and populations of cats. For example, among cats presented to veterinarians, the prevalence of FeLV viraemia (RT-qPCR) varies from 0 to 8% across Europe [3], and FIV seroprevalence varies from 2.5% to >27% worldwide [4]. 

In Hong Kong, cats presented to veterinarians can be broadly considered either community cats, i.e., free-roaming cats living in colonies that are cared for by members of the public, or are client owned, with some movement of cats between these categories. Understanding the epidemiology of FeLV and FIV in these clinically-relevant populations will support decision making by local veterinarians, as well as contribute to the global evidence base for these important feline pathogens.

Domestic cat hepadnavirus (DCH) is a novel, hepatitis-B-like virus first reported in Australia in 2018 [5], and subsequently detected in cats in Europe, Asia, and the USA [6,7,8]. Based on the pathobiology of hepatitis-B viruses that infect humans and rodents, a pathogenic role for DCH in some feline liver diseases is possible [9,10]. DCH DNA has been detected in hepatocellular carcinoma and chronic hepatitis in cats using PCR and in situ hybridization [11,12]. Further studies of spontaneous disease will help to clarify whether DCH plays an aetiological role in specific pathologies. The reported molecular prevalence of DCH varies from <1% to >18% [6,7,8]. In Hong Kong, DCH DNA has been detected in 57/513 (11.1%) of client-owned cats [13], but data for community cats in the region are not yet available. 

The aims of this study were to investigate the frequency and potential risk factors for (i) FeLV and FIV among community and client-owned cats and (ii) the molecular detection of DCH among community cats in Hong Kong.

## 2. Materials and Methods

### 2.1. Ethics Statement 

This study was approved by the Animal Research Ethics Committee of the City University of Hong Kong (A-0696 and A0478). 

### 2.2. Populations Sampled and Data Collection

Two populations of cats were investigated in a cross-sectional observational study, community cats and client-owned cats. 

#### 2.2.1. Community Cats 

Whole blood (EDTA and clotted) was obtained with owner consent from community cats enrolled in a trap–neuter–return (TnR) program with the Society for the Prevention of Cruelty to Animals, Hong Kong, between January 2021 and May 2021. Separated serum, and whole EDTA blood was stored frozen at −80 °C until testing. 

#### 2.2.2. Client-Owned Cats

Residual diagnostic EDTA blood was obtained with owner consent from cats presenting to a multi-specialist and primary accession hospital (CityU Veterinary Medical Centre, Hong Kong), between June 2020 and September 2021. 

Data on the source, age, sex, breed, and neuter status were recorded. Plasma was separated and the remaining sample was stored at −80 °C. 

### 2.3. FeLV-Antigen and FIV-Antibody Testing

Serum or plasma from all samples was tested for FeLV p27 antigen and for antibodies recognising FIV p15 and p24 using SNAP FIV/FeLV combo (IDEXX, Westbrook, ME, USA) according to the manufacturer’s instructions. FeLV p27 Snap-positive samples were further analysed for free p27 using sandwich enzyme-linked immunosorbent assay (ELISA), as described previously [14]. All samples were tested in duplicate and the absorbances were read using a microplate reader (Synergy H1, Biotek, VT, USA). Values > 20% of the positive control were considered to be positive [15].

### 2.4. Molecular Testing for FeLV

DNA was extracted from 100 µL of thawed whole blood or vortexed blood clots using the DNeasy Blood and Tissue kit (Qiagen GmbH, Hilden, Germany) and an elution volume of 50 µL. Extracted DNA from all samples was shipped on dry ice to the Clinical Laboratory, University of Zurich, Switzerland, where real-time quantitative PCR (qPCR) testing amplifying part of U3 long terminal repeat was performed as described previously [16] with some modifications. Proviral DNA was amplified from 5 μL of DNA in a 25 μL reaction containing the DNA quantitative PCR Mastermix (Eurogentec, Seraing, Belgium), 480 nM primers (FeLV_U3-exo_f, FeLV_U3-exo_r), and a 160 nM probe (FeLV_U3_probe). All oligonucleotides were synthesized using Microsynth AG (Balgach, Switzerland). The temperature profile consisted of 2 min at 50 °C, denaturation for 10 min at 95 °C, followed by 45 cycles of 95 °C for 15 s, and 60 °C for 1 min. The FeLV proviral copy numbers in the single samples were determined by co-amplifying 10-fold serial dilutions of a DNA standard template, as described previously [16]. Samples with three or more copies per reaction were considered positive. 

In addition, a real-time PCR targeting a conserved region in FeLV gag was performed as described previously [17]. PCR amplification using the forward primer G1-1515F (5′-CAACAACCGACCCCAGTATT-3′) in a concentration of 0.3 µM and the reverse primer G1-1611R (5′-AGTTAGGGCCACTGGATCTT-3′) in a concentration of 0.9 µM resulted in a PCR product of 97 bp. The probe (5′-CAGCTTCAGACTTGTATAACTGGAAGTCGCA-3′) was labelled at the 5′-end with the fluorescent reporter dye FAM and at the 3′-end with the dark quencher dye BHQ-1. DNA was amplified using an ABI Prism 7500Fast sequence detection system using TaqMan^®^ Fast Universal PCR Master Mix (Applied Biosystems, Thermo Fisher Scientific, Waltham, MA, USA). The temperature profile consisted of a denaturation for 2 min at 95 °C, followed by 45 cycles of 94 °C for 3 s, and at 60 °C for 30 s.

Finally, two nested (n) PCRs amplifying FeLV, either a conserved part of the U3 LTR, or a region between U3 and *gag*, yielding second round PCR products of 166 bp or 601 bp respectively)were performed on qPCR-positive samples, as described previously [18,19]. 

### 2.5. Real-Time Quantitative PCR for DCH Detection in Community Cats

A subset of samples that had sufficient sample remaining were tested for DCH using qPCR as described previously [20]. In brief, 25 μL reactions were prepared comprising 100 ng template DNA or 10 μL of plasmid standard (containing a 1.4 kb fragment of the polymerase region of the Australian reference strain AUS/2016/Sydney), and 12.5 μL of master mix (IQ Supermix; Bio-Rad Pacific Limited, Hong Kong), containing 600 nM of each primer and 200 nM of probe. Thermal cycling consisted of activation of Taq DNA polymerase at 95 °C for 3 min, and then 42 cycles of denaturation at 95 °C for 10 s, and annealing-extension at 60 °C for 30 s. A sample was defined as positive if ≥10 copies of DCH DNA were detected in ≥ two (of three) replicates with a Ct value of ≥38.5. The cut-off for R-squared was 0.980, and for efficiency was 90–110%. 

### 2.6. Statistical Analyses

All data management and analyses were performed using Stata v17 (Stata Corp LLC, College Station, TX, USA). Age was categorized at as ≤2 or >2 years, and breed was categorized as pure or mixed for risk factor analyses. Descriptive statistics were generated for all variables. Where data allowed, the univariable associations between the independent variables of interest (e.g., source, sex, age, breed) and the outcomes of interest (seropositivity to FIV and DCH DNA detection) were evaluated using a chi-squared test, with the significance level set at 0.05. 

## 3. Results

### 3.1. Population Characteristics

**Figure 1:**
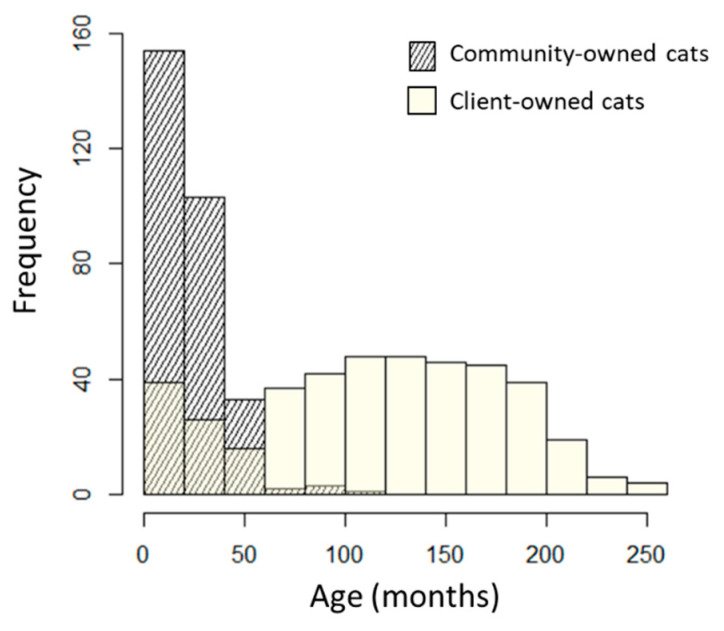
Age distribution of 298 community cats and 415 client-owned cats sampled in Hong Kong.

In total, 713 individuals were available for study, comprising 298 community cats and 415 client-owned cats. The characteristics of each population are presented in Table 1 and the age distributions in Figure 1. Community cats were predominantly young (median age 18 months, range 6–96 months, interquartile range [IQR] 13 months), had a male: female ratio of 0.75:1, were entirely mixed bred, and were sexually intact, except for 6 cats (4 females, 2 males) that were found to have already been neutered after trapping. In contrast, client-owned cats were an older population (median age 120 months, range 2–254 months, interquartile range 92 months) with a greater proportion of male cats (male: female ratio 1.71:1), purebred, and neutered cats than the community cats.

### 3.2. FIV Serology

FIV seroprevalence in community cats (46/298, 15.4%) was significantly higher than that in client-owned cats (13/415, 3.1%) (*p* < 0.001). The median age of FIV-seropositive cats was 24 months (IQR 18–36 months) for community cats and 111 months (IQR 51–156 months) for client-owned cats. The results of univariable analyses are presented in Table 2. Neither age nor sex was associated with FIV seropositivity. Among client-owned cats, mixed breed was a risk factor for FIV seropositivity compared with purebred. Community cats included only one purebred cat hence examination of breed as a risk factor was not relevant for this population.

### 3.3. FeLV-Antigen and Proviral-DNA Detection

Among community cats, neither FeLV antigen nor FeLV proviral DNA were detected in any of the 298 cats tested. Antigen testing was positive for 5/415 client-owned cats but FeLV infection was not confirmed using qPCR (U3 and gag) in any of these five cats or using the nested U3 PCR; however, three of the five cats tested also positive (>20%) and an additional cat questionably positive (>4%, recommendation to retest at a later timepoint) in the p27 ELISA. A single client-owned cat, a 6-year-old male-neutered Persian, yielded a positive result for FeLV U3 qPCR at 14 copies/reaction. 

### 3.4. Domestic Cat Hepadnavirus DNA Detection in Community Cats

DCH DNA was detected in 34/193 community cats (17.6%). The median viral load was 6.32 × 10^3^ copies/reaction and the range for the 5th to the 95th percentile of this population was 4.72 × 10^2^ to 1.09 × 10^9^ copies per mL. None of age, sex, or FIV infection were associated with DCH detection (Table 3).

## 4. Discussion

To the authors’ knowledge this is the first study to investigate the epidemiology of retrovirus infections in cats in Hong Kong. The low frequency of FeLV detection with only 1 of 713 cats testing positive on qPCR, carries important implications for the interpretation of antigen testing in the region. Testing for p27 antigen is the recommended screening test for FeLV, and positive results should be confirmed with qPCR [21]. Repeat testing for dual (antigen and qPCR) positive cats is indicated because many cats suppress virus replication and subsequently test negative for p27 antigen (within 16 weeks, but often sooner) [18]. These regressively infected cats do not completely eliminate the virus and may still test positive on qPCR, usually with a low viral load. Regressively infected cats are neither at risk from FeLV-related diseases nor a source of new infections, unless the infection is reactivated [15,22,23]. However, cats that persistently test antigen/qPCR positive represent clinically-relevant, progressive infections [24]. 

The single purebred, client-owned cat testing positive for FeLV qPCR was antigen negative. No further information was available for this cat. Regressive infection is the most likely explanation for this result, and the low viral load in this sample is consistent with this explanation. Early FeLV infection is also possible since cats exposed to FeLV can test qPCR positive within a few days, but may take weeks to test positive for antigens. We were unable to obtain sequence data from the qPCR-positive cat using nPCR, likely because of the low viral load. Whether FeLV infection might be more common among sick cats in Hong Kong was not investigated here; although, of 210 samples in total submitted to CityU Veterinary Diagnostic laboratory for FeLV RT-qPCR from 21 May 2018 to 31 May 2023, only one sample tested positive (Fraser Hill, Arthur Cheng, personal communication). The case was a 7 yo MN purbred cat believed to have been imported into Hong Kong. The result was confirmed with nPCR and sequencing in our lab (Genbank accession number: OR876264). This supports our finding that FeLV infection is rare in Hong Kong. 

In our study, 5 of 713 cats (0.07%) tested positive for FeLV antigen, but negative for provirus using qPCR and nPCR. This combination of results can indicate focal infection, characterised by persistent or intermittent antigenaemia without viraemia, early infection (although molecular tests are usually positive before antigen tests) or a “false-positive” antigen test result [24,25]. Without additional information, such as the results of anti-FeLV-antibody testing to investigate exposure, patient risk profile, and repeat testing, it is not possible to definitively distinguish between these scenarios. However, the very low prevalence of molecularly confirmed FeLV infection detected in this study supports “false-positive” antigen test results as being the most likely explanation for these FeLV-antigen-positive, provirus-negative results. This is not a reflection of the capabilities of the POC test used in this study. In fact, the diagnostic specificity of the SNAP FIV/FeLV combo has been shown to bevery high in other cat populations at 98.2% [26] and 97.3% [27] compared with virus isolation and 100% using qPCR as the gold standard [28,29]. However, the very low prevalence of FeLV detection in cats in Hong Kong also affects the specificity of the tests used [30], and it means that the positive predictive value (PPV) of antigen testing is low because the frequency of false positive exceeds that of true positive results. Confirmation of positive FeLV-antigen tests with qPCR should be considered essential in Hong Kong. FeLV-antigen testing is still appropriate as a first-line screening test for the following reasons; negative results are reliable; clinically relevant FeLV infections (i.e., cats that shed virus and succumb to FeLV-related diseases) test concurrently antigen positive and qPCR positive; and the results of qPCR alone can be difficult to interpret. Setting owners’ expectations that several tests may be required to determine the cat’s true FeLV status is especially useful in regions of low FeLV prevalence such as Hong Kong.

Interpretation of FIV serology for an individual cat considers individual infection risk as well as test performance to inform whether additional testing is required to determine infection status [21]. In this population, where individual assessment was not feasible, a positive serology result for FIV was considered to be a marker of FIV infection for reasons outlined here. The reported specificity of the POC test used in this study for FIV antibody detection is 99% compared with virus isolation [29]. Potential interference with FIV serology from maternally-derived antibodies, which can persist up to 6 months of age, was minimised. The youngest cats testing seropositive for FIV were community cats estimated to be 6 months and 10 month-old. It is conceivable that maternal antibodies could account for the FIV seropositive result in the younger cat only. The FIV vaccine, Fel-O-Vax^®^ FIV (Boehringer Ingelheim, North Ryde, NSW, Australia), has never been routinely available in Hong Kong, minimizing potential interference from vaccine-induced antibodies. Full vaccination and travel histories for individual cats were not available, so vaccine-associated interreference with FIV serology, like potential interference from maternal antibodies, is not completely eliminated. The reported sensitivity of the POC test for FIV-antibody detection, again using virus isolation as the gold standard, is 97.9% [29]. Molecular testing alongside serology may have identified FIV PCR-positive/seronegative cats. This is reported to be a rare scenario but may occur in in some cases of advanced disease [31]. In addition, FIV sequence variation could potentially influence the diagnostic sensitivity of serologic tests [32]. However, the POC test utilizes two conserved target antigens, p15 and p24, reducing the likelihood that sequence variation could result sufficient antigenic variation to result in negative serology in cats exposed to FIV 

FIV prevalence was significantly higher in community cats (15.4%) than client-owned cats (3.1%). This result is not unexpected since being sexually intact and having outdoor access, both more common in the community cats, are well-documented risk factors for FIV infection [33,34,35,36]. Testing of community cats pesented for TnR selected for a young subpopulation, so FIV seroprevalence in the broader community-cat population may actually be higher than the 15.4% observed because the risk of acquiring FIV infection increases with age. Similar studies testing cats enrolled in TnR programs in other regions reported lower FIV seroprevalences than that observed here, at 6.4% and 3.3% in the USA, 6.6% in Italy, and 1% in Spain [37,38,39,40]. Nonetheless, small studies of free-roaming, unowned cats in Rio de Janeiro, Brazil, and Southern Vietnam reported FIV seropositive rates higher than observed here at >50% [41] and 22% [42], respectively. Male sex is a consistent risk factor for FIV infection in previous studies [35,36,43]. This common epidemiological finding supports the assertion that transmission of FIV by inoculation of virus in saliva during territorial aggression is the most common natural route of transmission. Sex was not identified as a risk factor in our study. Among client-owned cats, the low number of FIV seropositive cats might explain this observation, but the absence of a sex-predisposition in community cats is intriguing. Being sexually intact, which 98% of community cats were at testing, is also a risk factor for FIV but this has not masked sex predisposition in other studies of TnR cats [44]. Husbandry practices for community cats in Hong Kong might reduce competition between males and females for resources such as food. The absence of a sex predisposition for FIV has been reported previously in cats kept in similar circumstances in Singapore [45]. Vertical transmission of virus might also contribute to equal representation of males and females among FIV-seropositive cats, something that could be investigated in the future. The influence of different virus strains on regional epidemiology is unknown. A recent global overview of feline virus sequences highlights the potential to consider FIV sequence comparisons between regions as a factor affecting local virus transmission characteristics [46]. No sequences are yet available for FIV from Hong Kong, so this analysis was not possible here. 

This study complements a recent study of the molecular epidemiology and phylogeny of DCH in client-owned cats in Hong Kong and demonstrates that DCH is also circulating among community cats [13]. Risk factors for DCH viraemia were not identified here. Detection of DCH DNA confirms infection, but a negative qPCR result does not rule out infection since not all infected cats are expected to be viraemic [47]. Progress has been made towards developing a panel of markers that will help to clarify the threat that DCH poses to feline health, as well as its natural history and global epidemiology [48]. 

## 5. Conclusions

Hong Kong is a region of low FeLV prevalence, so positive antigen test results should be confirmed with qPCR. FIV infection is common in community cats, and the absence of a sex predisposition, seen previously in cats managed similarly, raises questions about virus-transmission dynamics in these groups. DCH infection is very common in Hong Kong, both in client-owned and community cats, highlighting the importance of understanding the pathogenic potential of this virus for cats.

## Figures and Tables

**Table 1:** Population characteristics of 298 community and 415 client-owned cats sampled in Hong Kong.

Variable	Category	Community Cats (% of 298)	Client-Owned Cats (% of 415)
Sex	Male	128 (43%)	262 (63.1%)
	Female	170 (57%)	153 (36.9%)
Neuter status	Neutered	6 (2%)	169 (40.7%)
	Entire	292 (98%)	246 (59.3%)
Breed	Purebred	0 (0%)	230 (55.4%)
	Mixed bred	298 (100))	185 (44.6%)

**Table 2:** Results of FIV serology and their association with independent variables of interest among community and client-owned cats in Hong Kong.

Variable	Category	Community Cats (n = 298)		Client-Owned Cats (n = 415)	
		FIV Seropositive (%)	*p* Value	FIV Seropositive (%)	*p* Value
Age	≤2 years old	30/221 (13.6%)	0.099	1/45 (2.2%)	0.710
	>2 years old	16/74 (21.6%)		12/370 (3.2%)	
Sex	Female	22/170 (12.9%)	0.169	5/153 (3.3%)	0.904
	Male	24/128 (18.8%)		8/262 (3.1%)	
Breed	Mixed breed	46/297 (15.5%)	NA ^^^	11/185 (5.9%)	**0.004**
	Purebred	0/1 (0.0%)		2/230 (0.9%)	

^^^ Robust statistical comparison was not possible.

**Table 3:** **Detection frequency** of DCH DNA in blood from 193 community cats in Hong Kong.

Variable	Category	DCH DNA Positive (%)	*p* Value
Age	≤2 years old	25/147 (17.0%)	0.691
	>2 years old	9/46 (19.6%)	
Sex	Female	17/108 (15.7%)	0.441
	Male	17/85 (20.0%)	
FIV status	Positive	31/170 (18.2%)	0.540
	Negative	3/23 (8.7%)	

## Data Availability

Data are contained within the article.
